# Performance measures of the medical priority dispatch system in an urban basic life support system

**DOI:** 10.1186/s13049-025-01410-6

**Published:** 2025-05-21

**Authors:** Vittorio Nicoletta, Maxime Robitaille-Fortin, Valérie Bélanger, Éric Mercier, Jessica Harrisson

**Affiliations:** 1 Department of Logistics and Operations Management, HEC Montréal, 3000 Côte-Sainte-Catherine Road, Montreal, QC H3T 2A7 Canada; 2 Canadian Forest Service, Laurentian Forestry Centre, Natural Resources Canada, Quebec City, Canada; 3 Coopérative des techniciens ambulanciers du Québec (CTAQ), Quebec City, Canada; 4 VITAM – Centre de recherche en santé durable, Université Laval, Quebec City, Canada; 5 Coordination nationale de l’accès aux soins et services, Santé Québec, Quebec City, Canada; 6 Faculty of Medicine and Health Sciences, Université de Sherbrooke, Sherbrooke, Canada

**Keywords:** Emergency medical services, Triage, Paramedic, Ambulance, Data analysis

## Abstract

**Background:**

Accurate dispatch prioritization for emergency medical services (EMS) is essential for optimizing resource allocation and ensuring timely emergency response. In the Province of Quebec, Canada, a locally adapted dispatch system was implemented using the standardized codes of the Medical Priority Dispatch System (MPDS) but with regional priority definitions. Despite periodic reviews, the system’s performance has not been formally assessed. This study evaluates the effectiveness of this prioritization system by comparing priority levels assigned at call-taking with on-scene paramedic assessments and by examining how the system’s performance has evolved over three years and across chief complaints.

**Methods:**

In this retrospective observational study, we analyzed EMS dispatches in the Capitale-Nationale administrative region of the Province of Quebec, Canada, between July 15 and December 15 over three consecutive years (2021, 2022, and 2023). We assessed system performance using sensitivity, specificity, overtriage, undertriage, predictive values, and accuracy. Statistical analyses included chi-square tests for priority consistency and pairwise t-tests for performance changes over time. Additionally, we examined variations across chief complaints to identify high overtriage and undertriage medical conditions.

**Results:**

This study analyzed 96,099 EMS dispatches over a three-year period. While 61.8% of these dispatches were classified as urgent at call-taking, paramedics later determined that 79.7% of all cases were stable and required non-urgent transport, indicating a high level of overtriage. Conditions such as abdominal pain, falls, and psychiatric issues were the chief complaints that showed high overtriage rates (> 90%), whereas allergic reactions, diabetic problems, and heart conditions had the highest undertriage rates (> 10%). Over the three-year period, priority modifications led to a 2.5% decrease in undertriage but a 3.7% increase in overtriage (*p* < 0.05), highlighting the ongoing challenge of balancing accuracy with an adequate response in dispatch prioritization.

**Conclusion:**

The studied prioritization system effectively identifies non-urgent dispatches but exhibits a high overtriage rate, which strains EMS resources. The recent priority modifications further increased overtriage, underscoring the challenge of balancing resource allocation with timely intervention. Refining dispatch criteria and integrating secondary triage or AI-based decision support could potentially improve accuracy and system efficiency.

**Supplementary Information:**

The online version contains supplementary material available at 10.1186/s13049-025-01410-6.

## Background

Dispatching systems are a critical component of emergency medical services (EMS) as they play a pivotal role in prioritizing and allocating resources to patients based on urgency and need. These systems directly impact the availability of prehospital resources and the overall efficiency of EMS operations [1]. Accurate dispatching is essential to prevent overtriage, in which resources are unnecessarily deployed due to an overestimation of patient needs. The result of overtriage is an overburdening of EMS capacity and reduced availability for true emergencies [2, 3]. Conversely, undertriage, or the underestimation of patient needs, can delay care for critically ill patients and subsequently lead to significant risks to patient safety and outcomes [4]. Robust and reliable EMS dispatch and triage systems that efficiently allocate resources and provide timely emergency response are therefore essential for the effective functioning of prehospital care [5].

EMS dispatching systems typically fall into two main categories: the Medical Priority Dispatch System (MPDS) [6], which is widely adopted in English-speaking countries, and Criteria Based Dispatch (CBD) [7], which is prevalent in European countries, especially Nordic ones. The MPDS employs standardized scripted questions and codes, while CBD relies on the dispatcher’s experience and judgment to assess calls. Both systems aim to accurately match EMS resources (basic life support, advanced life support, and first responders) with the priority and specific needs of each call. Despite the widespread use of EMS dispatch systems, few studies have rigorously evaluated their accuracy, and the findings of those that do exist varied significantly depending on the system and context [8–10].

In the early 1990s, EMS organizations in the Province of Quebec, Canada, adopted the MPDS to identify chief complaints and determine dispatch priorities and responses [11]. Few years later, provincial authorities opted to develop their own prioritization system but maintained the flexibility to adjust priority levels and tailor call prioritization and response time targets to regional needs. This means that the system identifies chief complaints using the MPDS code system but uses locally adapted priority criteria to define dispatch priorities and responses. Since the time of the system’s implementation, local priorities associated with MPDS codes have been regularly reviewed by a provincial committee of experts, with the latest modifications made in June 2023. However, no studies have compared the performance of this locally adapted system with that of standard MPDS recommended dispatch priorities or evaluated whether its effectiveness has increased or decreased as a result of the changes.

To evaluate the performance of the EMS priority system used in the Province of Quebec, Canada, we compared priority levels assigned at call-taking with on-scene paramedic assessments. Specifically, we assessed overtriage, undertriage, sensitivity, specificity, predictive values, and overall accuracy to determine the system’s effectiveness in identifying patient acuity. We analyzed three years of data to examine the impact of changes to the prioritization system and assessed whether these modifications have influenced performance. We also explored variations across different chief complaints and identified specific areas for improvement.

## Methods

### Study setting

This study was conducted in the Capitale-Nationale administrative region of Quebec, Canada. This territory of over 20,000 km^2^ includes urban, suburban and rural areas and has a population of 770,000 [12]. In the Province of Quebec, Canada, prehospital care is regionalized, with each region having its own communication centre that handles all calls and one or more EMS organizations that provide paramedical services. The Capitale-Nationale region is served by approximately 600 basic life-support paramedics, with each ambulance staffed by two paramedics. Annually, these paramedical teams are dispatched to about 70,000 calls [12]. The region also has ten emergency departments, including specialized centres (adult trauma centre, pediatric trauma centre, burn centre, cardiac institute) that serve the eastern part of the province (1–14).

Once a call is received at the communication centre, EMS dispatchers follow a computer-based, scripted algorithm (MPDS v13.3). Using the MPDS, the call is assigned a determinant code that is standardized across all MPDS users. This code consists of a number (code number 1 to 33) representing the type of medical emergency (or the chief complaint) and a letter (A–E) indicating the severity of the call and the associated response. Calls classified as Echo (E) are the most severe, followed by Delta (D), Charlie (D), Bravo (B), and Alpha (A). For example, 6D corresponds to breathing problems with severe distress, for which the MPDS advises dispatching an EMS unit with lights and sirens.

In Quebec, the MPDS determinant codes are translated into a locally adapted priority system, referred to as the P-system, to determine the appropriate response. In this system, P0 represents an immediate life-threatening emergency, such as cardiac arrest, which requires the fastest possible intervention. P1 includes high-priority emergencies that demand rapid response, such as heart attack or stroke. For both P0 and P1, ambulances must always travel to the patient with lights and sirens. P3 applies to urgent but stable cases that require timely medical attention, such as respiratory distress without severe impairment or severe pain. In these cases, paramedics have the discretion to use lights and sirens based on traffic conditions. P4 includes less urgent cases that do not require immediate intervention, such as a sick person feeling weaker than usual. Finally, P7 is designated for non-urgent situations or cases that could be referred to other healthcare services, such as a fall with minor extremity injuries. For P4 and P7 calls, the use of lights and sirens is not permitted. P2, P5, P6, and P8 are reserved for interfacility transports, which fall outside the scope of this study.

Upon arriving at the scene, the paramedics assess the patient and provide necessary care in accordance with established protocols and procedures. The goal is to stabilize the patient on scene before quickly transferring them to the appropriate facility for definitive care. Before transport, the paramedics assess the patient’s stability using a set of predefined criteria defined by the provincial committee of experts (Table 1) to determine whether urgent or immediate transport is required. Urgent transport is required when a patient is in an unstable clinical state and needs immediate medical attention. In such cases, the ambulance must proceed without delay, with lights and sirens activated so that the team can arrive as quickly as possible at the receiving facility. Immediate transport applies to potentially unstable situations that require rapid intervention and prompt departure. The team will drive in a non-emergency manner but may switch to emergency driving (lights and/or sirens) if an unexpected delay significantly impacts arrival time. Non-emergency transport is for stable patients and does not involve emergency driving.

**Table 1:** Criteria for on-scene paramedic assessment

Unstable patient (Return transport mode: urgent)	Potentially unstable patient (Return transport mode: immediate)	Stable patient (Return transport mode: non-urgent)
Hypotension (adult: systolic BP < 100) Cyanosis Diaphoresis Respiratory distress/insufficiency Altered level of consciousness (“V”, “P”, or “U”) Significant alteration in pulse (adult: < 50/min or > 150/min) Significant alteration in respiratory rate (adult: < 8/min or > 36/min) Significant alteration in oxygen saturation (< 85%)	Chest pain in patients over 35 years old Digestive hemorrhage with normal vital signs Vaginal bleeding (1 pad or more every 15 min) Severe and/or sudden headache with or without a history of syncope Acute neurological dysfunction (confusion, paralysis, paresis)	Any other conditions

### Study design

This retrospective observational study was conducted using data from all EMS dispatches in the Capitale-Nationale region between July 15 and December 15 over three consecutive years (2021, 2022, and 2023). Specifically, it includes dispatches from July 15 to December 15, 2021, July 15 to December 15, 2022, and July 15 to December 15, 2023. The data for this study were extracted from the electronic patient care record system. Each entry corresponds to a single patient and includes detailed information such as the time of the 911 call, sociodemographic details, MPDS code, priority level, ambulance dispatch time, ambulance arrival time, and destination hospital.

The priority levels assigned by the P-system were compared to on-scene assessments, based on the paramedics’ decision to perform either urgent or non-urgent transport, as per a methodology similar to that described in [8, 13]. For this analysis, both urgent and immediate return transports were classified as urgent, as they involve time-sensitive interventions requiring prompt medical response. In the P-system, P0, P1, and P3 are categorized as urgent dispatches, whereas P4 and P7 are classified as non-urgent. For comparison, in MPDS, E, D, and C dispatches are considered urgent, while B and A are non-urgent [14].

### Statistics

We evaluated the performance of the prioritization system using key performance metrics, including sensitivity, specificity, positive predictive value (PPV), negative predictive value (NPV), overtriage, undertriage, and accuracy (Table 2). These metrics were calculated using a crosstabulation of true positive (TP), false positive (FP), true negative (TN), and false negative (FN) values and reported as percentages with 95% confidence intervals. Chi-square tests were used to analyze the consistency between priority assessments. These indicators are widely used for evaluating prediction systems [4], including EMS prioritization [8, 15]. Additionally, we calculated the area under the curve (AUC) and have provided receiver operating characteristic (ROC) curves, which illustrate the trade-off between sensitivity and specificity.

**Table 2:** Measures used to evaluate the performance of the dispatching system - Adapted from [14]

		Paramedic assessment		
		Urgent (time-sensitive)	Non-urgent (less-time critical)	
**Priority at call-taking**	**Urgent**	True positive (TP)	False positive (FP)	PPV = TP/(TP + FP)
	**Non-urgent**	False negative (FN)	True negative (TN)	NPV = TN/(FN + TN)
		Sensitivity = TP/(TP + FN)	Specificity = TN/(FP + TN)	Accuracy = (TP + TN)/ (TP + TN + FP + FN)
		Undertriage = FN/(TP + FN)	Overtriage = FP/(FP + TN)	

To assess the impact of the change made in June 2023 based on recommendations from the provincial committee of experts, we analyzed each year independently. A subgroup analysis was conducted excluding P3. Pairwise t-tests were conducted to evaluate consistency over the three years and to identify statistically significant differences in performance across time periods. Additionally, dispatches were grouped by chief complaint, and performance metrics were calculated for each medical condition with more than 100 dispatches. This approach was used to identify specific dispatch categories that contributed to increased overtriage or undertriage and to pinpoint areas requiring further investigation or potential system adjustments.

All analyses were conducted using R [16], with the *caret* and *pROC* packages. The initial analysis was performed by an analyst and subsequently validated by the rest of the team.

### Ethics

This study was approved by the HEC Montréal ethics committee. Individual patient consent was deemed unnecessary.

## Results

All emergency dispatches that resulted in patient transport to a hospital in the region during these periods were considered for inclusion (*n* = 156,197). Calls related to interfacility transports or police/firefighter assistance were excluded (*n* = 23,836). Additionally, calls that were cancelled or that resulted in the patient refusing transport were excluded (*n* = 30,971). We also excluded calls with missing data related to the MPDS code, priority code, or paramedic assessment (*n* = 858). Finally, duplicate entries were removed from the dataset (*n* = 4,433). As decided in consultation with the research team, entries were considered duplicates if they shared the same call number and had matching patient sociodemographic information (age and gender). A total of 96,099 EMS dispatches were considered for analysis, with a similar exclusion rate for the three periods.

### Priority assessment at call-taking compared to on-site paramedic assessment

Table 3 compares the priority level assigned at call-taking using the P-system and the on-scene paramedic assessment. We performed multiple chi-square tests to analyze the consistency between the priority assessments (one for each year). Each test returned a p-value < 0.001, corresponding to a statistically significant difference between the priority at call-taking and the paramedic assessment.

Overall, the majority of dispatches were classified as urgent, with 40.9% (*n* = 39,248) categorized as P0 or P1 and 38.1% (*n* = 36,641) as P3. In contrast, only 21% (*n* = 20,210) were classified as non-urgent (P4 or P7) at call-taking. Out of all of these dispatches, paramedics later determined that 79.7% (*n* = 76,612) of patients were stable upon assessment and subsequently required a non-urgent return transport. A detailed analysis reveals that, among all dispatches initially classified as P0, 51% were ultimately deemed non-time-sensitive (non-urgent) by paramedics. This proportion increases to 67.5% for P1 and 85.9% for P3, indicating that a significant percentage of dispatches initially prioritized as urgent were considered non-urgent upon paramedic assessment.

**Table 3:** Number of calls by priority at call-taking and paramedic assessment

Priority at call-taking	Paramedic assessment		Total
	Unstable or potentially unstable (Return transport mode: Immediate & Urgent)	Stable (Return transport mode: Non-urgent)	
**P0**	1,453 (49%)	1,517 (51%)	2,970
**P1**	11,805 (32.5%)	24,473 (67.5%)	36,278
**P3**	5,147 (14.1%)	31,494 (85.9%)	36,641
**P4**	556 (6.3%)	8,323 (93.7%)	8,879
**P7**	526 (4.7%)	10,805 (95.3%)	11,331
**Total**	19,487	76,612	96,099

Percentages in parentheses are relative to the total for each P-system priority level

### Performance of the prioritization system and changes in performance over time

**Fig. 1:**
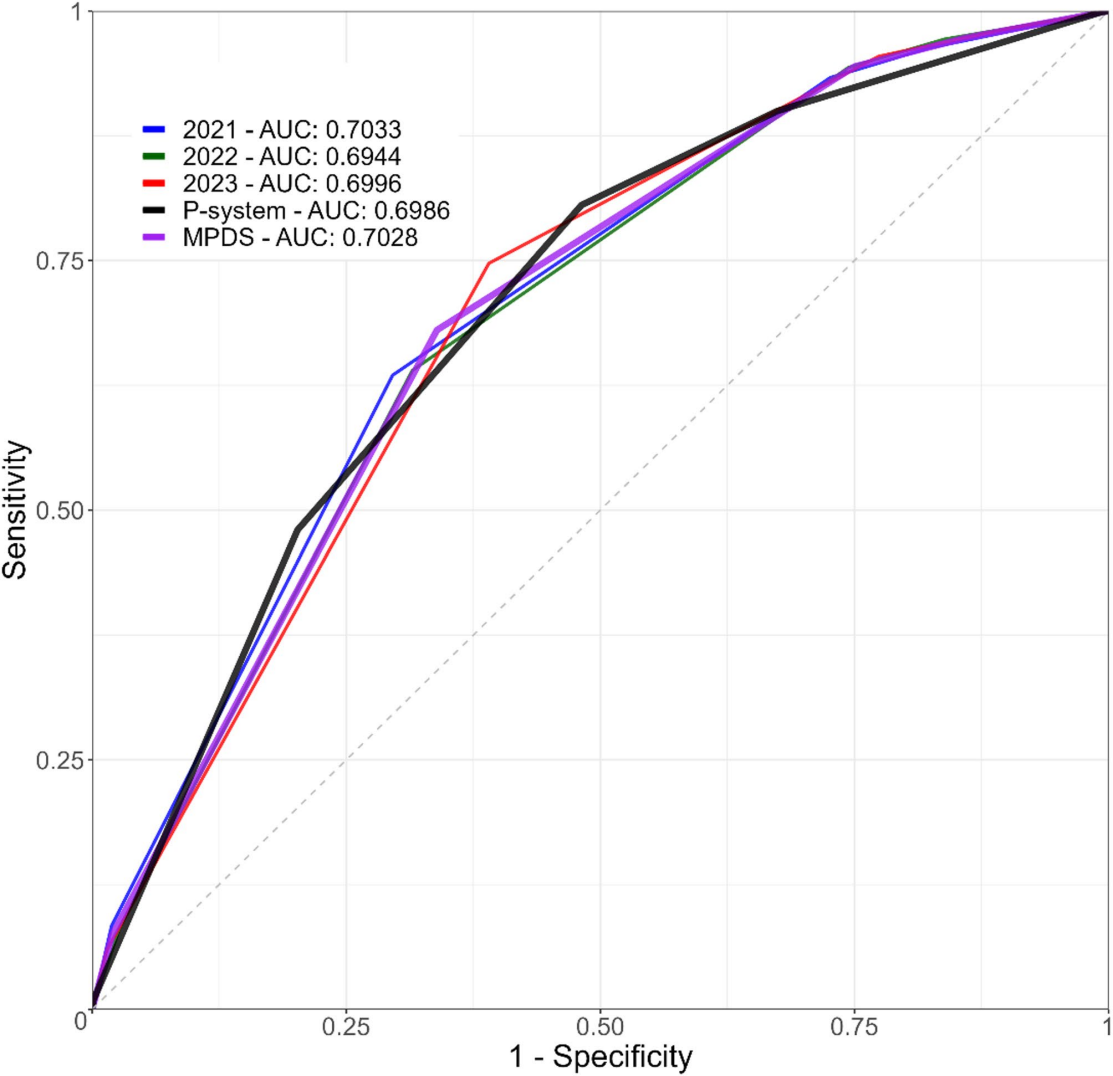
ROC curves for 2021, 2022, 2023, overall P-system, and overall MPDS

Table 4 presents the performance evaluation of the P-system based on key metrics such as sensitivity, specificity, accuracy, undertriage, overtriage, NPV, and PPV across the entire dataset. For comparison, we also report the performance of MPDS when this system is used to determine dispatch priority and response levels according to MPDS standardized protocols (see Supplementary Material A for additional results). Figure 1 provides a visual comparison by illustrating ROC curves and AUC for both prioritization systems.

**Table 4:** Performance metrics for the P-system and MPDS system

Performance metric	*P*-system			*P*-system (2021–2023)	MPDS (2021–2023)	*P*-system* (2021–2023)
	2021	2022	2023			
**Sensitivity**	93.3%	94.4%	95.5%	**94.5%**	**90%**	**92.5%**
**Specificity**	27.4%	25.6%	22.6%	**25.0%**	**32.6%**	**42.4%**
**Undertriage**	5.9%	5.5%	4.7%	**5.4%**	**7.2%**	**5.4%**
**Overtriage**	75.5%	75.1%	77.7%	**75.7%**	**74.7%**	**66.2%**
**NPV**	94.1%	94.5%	95.3%	**94.6%**	**92.8%**	**94.6%**
**PPV**	24.5%	24.9%	22.3%	**24.3%**	**25.3%**	**33.8%**
**Accuracy**	40.9%	39.8%	37%	**39.1%**	**44.2%**	**54.5%**

* P3 excluded from the analysis

Overall, for P-system, the sensitivity is high at 94.5% (95% CI 94-94.8), along with an overtriage rate of 75.7% (95% CI 75.4–76) and a PPV of 24.3% (95% CI 24-24.6). Conversely, specificity is relatively low at 25.0% (95% CI 24.7–25.3), and undertriage and NPV are 5.4% (95% CI 5.1–5.7) and 94.6% (95% CI 94.3–94.9) respectively. The accuracy and the AUC are 39.1% (95% CI 38.8–39.4) and 0.6986 respectively. For the MPDS system, the sensitivity is 90.0% (95% CI 89.6–90.4), along with an overtriage rate of 74.7% (95% CI 74.3–75) and a PPV of 25.3% (95% CI 25-25.7). Specificity is 32.6% (95% CI 32.2–32.9), while undertriage and NPV are 7.2% (95% CI 6.9–7.5) and 92.8% (95% CI 92.4–93.2), respectively. The accuracy and the AUC are 44.2% (95% CI 43.9–44.5) and 0.7028. Practitioners recognize that P3 dispatches, which account for over 40% of total call volume, introduce significant heterogeneity and are difficult to classify definitively as either urgent or non-urgent. To assess their impact on system performance, we conducted all analyses by excluding P3 calls from the dataset. This approach allowed us to evaluate system performance considering only P0, P1, P4, and P7. The main results of this analysis are presented in the last column of Table 4, with detailed results available in Supplementary Material B. Since P3 dispatches are frequently overtriaged, their removal from the dataset increases specificity to 42.4% (95% CI: 41.9–42.9), PPV to 33.8% (95% CI: 33.3–34.2) and accuracy to 54.5% (95% CI: 54.1–54.9), while reducing the overtriage rate to 66.2% (95% CI: 65.8–66.7). Sensitivity slightly decreases to 92.5% (95% CI: 92–93), while other performance measures remain unchanged.

Finally, an analysis of system performance over three years revealed a shift in 2023 following modifications to the P-system. Priority levels were adjusted for 126 codes (8.3%), with 102 codes (6.7%) upgraded to a higher urgency level and 24 codes (1.6%) downgraded to a lower urgency level. These changes affected 6,263 calls, representing 16% of total call volume in 2023 (see Supplementary Material C for details). A pairwise comparison of performance metrics between 2021 and 2023 and 2022–2023 showed statistically significant differences (*p* < 0.05), highlighting variations over time. As a result, 2023 saw an increase in overtriage, sensitivity, and NPV, while specificity, undertriage, PPV, and accuracy decreased.

### Performance of the prioritization system across chief complaints

The analysis of performance consistency across chief complaints revealed variations in performance, and specific medical conditions were found to have a high overtriage rate. A total of 34 dispatch categories were analyzed, with 27 having more than 100 dispatches. The results in Tables 5 and 6 indicate that abdominal pain (*n* = 3,967), back pain (*n* = 1,886), falls (*n* = 14,380), and psychiatric problems (*n* = 3,441) were the most frequent call types associated with overtriage rates exceeding 90%. In contrast, allergic reactions (*n* = 837), diabetic problems (*n* = 532), heart problems (*n* = 1,876), and overdose/poisoning (*n* = 2,126) exhibited the highest undertriage rates (greater than 10%). Thirteen chief complaints had a sensitivity of 100%, while none had a specificity of 100%. Specificity was generally below 50%, except for back pain (62%), sick person (57%), and traumatic injuries. Similarly, PPV was typically below 50%, except for allergic reactions (53%) and cardiac arrest (73%).

**Table 5:** Differences between dispatch priority and paramedic assessment across chief complaints

Chief complaint (*n*)	Dispatch priority at call-taking % (*n*)		Paramedic assessment % (*n*) Urgent		Paramedic assessment % (*n*) Non-urgent	
	Urgent (P0, P1, P3)	Non-urgent (P4, P7)	TP	FP	TN	FN
Abdominal pain (3,967)	53 (2,106)	47 (1,861)	10 (220)	90 (1,186)	93 (1,738)	7 (123)
Allergic reaction (837)	87 (730)	13 (107)	53 (384)	47 (346)	82 (88)	18 (19)
Assault (489)	95 (464)	5 (25)	14 (64)	86 (400)	100 (25)	0 (0)
Back pain (1,886)	39 (728)	61 (1,158)	5 (39)	95 (689)	99 (1,143)	1 (15)
Breathing diff. (9,739)	100 (9,739)	0 (0)	37 (3,568)	63 (6,171)	NA (0)	NA (0)
Hazard exposure (114)	100 (114)	0 (0)	39 (44)	61 (70)	NA (0)	NA (0)
Cardiac arrest (103)	87 (90)	13 (13)	73 (66)	27 (24)	100 (13)	0 (0)
Chest pain (11,183)	100 (11,183)	0 (0)	30 (3,393)	70 (7,790)	NA (0)	NA (0)
Choking (371)	67 (248)	33 (123)	39 (96)	61 (152)	92 (113)	8 (10)
Convulsions (1,857)	100 (1,857)	0 (0)	40 (737)	60 (1,120)	NA (0)	NA (0)
Diabetic prob. (532)	70 (370)	30 (162)	32 (119)	68 (251)	84 (136)	16 (26)
Eye problems (110)	60 (66)	40 (44)	9 (6)	91 (60)	93 (41)	7 (3)
Falls (14,380)	74 (10,583)	26 (3,797)	10 (1,103)	90 (9,480)	93 (3,543)	7 (254)
Headache (1,082)	72 (776)	28 (306)	14 (109)	86 (667)	97 (296)	3 (10)
Heart problem (1,876)	96 (1,805)	4(71)	31 (558)	69 (1,247)	86 (61)	14 (10)
Hemorrhage (3,758)	87 (3,283)	13 (475)	14 (455)	86 (2,282)	95 (449)	5 (26)
Overdose / poison (2,126)	99 (2108)	1 (18)	31 (645)	69 (1,463)	89 (16)	11 (2)
Pregnancy (409)	97 (397)	3 (12)	47 (188)	53 (209)	100 (12)	0 (0)
Psychiatric (3,441)	71 (2,429)	29 (1,012)	8 (191)	92 (2,238)	97 (984)	3 (28)
Sick person (14,322)	46 (6,530)	54 (7,792)	14 (946)	86 (5,584)	94 (7,363)	6 (429)
Stroke (3,087)	100 (3,087)	0 (0)	35 (1,094)	65 (1,993)	NA (0)	NA (0)
Traffic incidents (3,644)	100 (3,644)	0(0)	23 (850)	77 (2,794)	NA (0)	NA (0)
Traumatic injuries (3,691)	52 (1,926)	48 (1,765)	15 (287)	85 (1,639)	97 (1,709)	3% (56)
Unconscious (7,750)	100 (7,750)	0 (0)	27 (2,077)	73 (5,673)	NA (0)	NA (0)
Unknown (966)	100 (966)	0 (0)	23 (221)	77 (745)	NA (0)	NA (0)
Palliative care (3,868)	64 (2,462)	36 (1,406)	31 (769)	69 (1,693)	95 (1,341)	5 (65)
Pandemic (128)	100 (128)	0 (0)	38 (49)	62 (79)	NA (0)	NA (0)

NA values not computed due to lack of sufficient emergency calls for the specified category

**Table 6:** Performance measures for each MPDS category by priority

Chief complaint (*n*)	Overtriage	Undertriage	Sensitivity	Specificity	PPV	NPV	Accuracy
**Abdominal pain (3**,**967)**	**90%**	7%	64%	48%	10%	93%	49%
*Allergic reaction (837)*	47%	*18%*	95%	20%	53%	82%	56%
Assault (489)	86%	0%	100%	6%	14%	100%	18%
**Back pain (1**,**886)**	**95%**	1%	72%	62%	5%	99%	63%
Breathing diff. (9,739)	63%	NA	100%	0%	37%	NA	37%
Hazard exposure (114)	61%	NA	100%	0%	39%	NA	39%
Cardiac arrest (103)	27%	0%	100%	35%	73%	100%	77%
Chest pain (11,183)	70%	NA	100%	0%	30%	NA	30%
Choking (371)	61%	8%	91%	43%	39%	92%	56%
Convulsions (1,857)	60%	NA	100%	0%	40%	NA	40%
*Diabetic prob. (532)*	68%	*16%*	82%	35%	32%	84%	48%
Eye problems (110)	91%	7%	67%	41%	9%	93%	43%
**Falls (14**,**380)**	**90%**	7%	81%	27%	10%	93%	32%
Headache (1,082)	86%	3%	92%	31%	14%	97%	37%
*Heart problem (1*,*876)*	69%	*14%*	98%	5%	31%	86%	33%
Hemorrhage (3,758)	86%	5%	95%	14%	14%	95%	24%
*Overdose / poison (2*,*126)*	69%	*11%*	100%	1%	31%	89%	31%
Pregnancy (409)	53%	0%	100%	5%	47%	100%	49%
**Psychiatric (3**,**441)**	**92%**	3%	87%	31%	8%	97%	34%
Sick person (14,322)	86%	6%	69%	57%	14%	94%	58%
Stroke (3,087)	65%	NA	100%	0%	35%	NA	35%
Traffic incidents (3,644)	77%	NA	100%	0%	23%	NA	23%
Traumatic injuries (3,691)	85%	3%	84%	51%	15%	97%	54%
Unconscious (7,750)	73%	NA	100%	0%	27%	NA	27%
Unknown (966)	77%	NA	100%	0%	23%	NA	23%
Palliative care (3,868)	69%	5%	92%	44%	31%	95%	55%
Pandemic (128)	62%	NA	100%	0%	38%	NA	38%

NA values not computed due to lack of sufficient emergency calls for the specified category

## Discussion

This study compares the priority established at call-taking with on-scene paramedic assessments and analyzes system performance over three years and across different chief complaints. Our study highlights critical challenges in EMS prioritization systems, as evidenced by the low specificity (25%) and high overtriage rate (74.5%) along with a undertriage over 5% observed in Quebec City from 2021 to 2023. A substantial proportion of ambulance dispatches classified as urgent (P1–P3) were ultimately deemed non-urgent by paramedics, with 86.1% of P3 dispatches classified as non-urgent based on the on-scene paramedic assessment. P3 cases pose a particular challenge, as they encompass a broad spectrum of conditions, making accurate triage difficult. Similar results are obtained for MPDS [17–19].

Our findings align with previous research on EMS prioritization systems (e.g., 8, 13, 14, 17 to 19). Similar to our study, Ball et al. [13] analyzed a three-level system that adapts MPDS determinants to local EMS priority systems. Their study found low time-critical accuracy for certain priority levels, particularly in high-acuity cases such as chest pain and seizures, when comparing dispatch decisions with paramedic assessments. Feldman et al. [14] evaluated MPDS in Toronto, Canada, by comparing dispatch priorities to the Canadian Triage and Acuity Scale (CTAS). Their findings revealed moderate sensitivity (68.2%) and specificity (66.2%), with performance variations across chief complaints. Furthermore, a recent literature review concluded that there is low-quality to very low-quality evidence supporting the accuracy of medical dispatching systems [10]. Collectively, these studies highlight persistent limitations in the ability of dispatch systems to reliably predict patient acuity across diverse EMS settings.

In Quebec, between 2021 and 2023, adjustments to priority classifications successfully reduced undertriage by 2.5% but led to a 3.7% increase in overtriage. These findings highlight the complexity of balancing overtriage and undertriage and the challenge of optimizing patient outcomes while ensuring system efficiency and safety. Determining the ideal balance remains a challenge, as there is no consensus in the literature on acceptable overtriage and undertriage thresholds [10, 13].

A closer examination of performance across chief complaints reveals considerable variation that is consistent with findings from previous studies [8, 13, 14, 20–22]. As observed in [8], we find that less-critical chief complaints, such as abdominal pain, back pain, general illness, falls, and psychiatric problems, tend to have higher specificity but also higher overtriage rates. In contrast, highly time-sensitive conditions, including chest pain, allergic reactions, stroke, heart problems, and cardiac arrest, exhibit high sensitivity to ensure that critical patients receive timely medical attention. While common in EMS prioritization systems as it reduces the risk of missing critically ill patients, overtriage also places a significant strain on these services. Excessive overtriage can delay responses to time-sensitive conditions and negatively impact paramedic well-being, which highlights the delicate balance required to optimize emergency dispatch accuracy.

The impact of overtriage extends beyond system inefficiencies and compromises operational performance and workforce sustainability. Overtriage impacts dispatch operations as it reduces dispatcher ability to allocate ambulances efficiently and increases the risk of “level 0” ambulance coverage, i.e. when no units are available to respond, leading to delays in treat critical patients. Additionally, urgent dispatch protocols often require paramedics to respond during breaks or at the end of their shifts, adding stress that can lower job satisfaction, increase turnover rates, and affect mental health [23]. Another consequence is the higher frequency of lights-and-sirens responses, which raises the risk of road accidents and poses safety concerns for both paramedics and the public [24]. Though less frequent, undertriage also remains a critical issue due to the delay in access to life-saving care for patients with time-sensitive conditions.

Improving prioritization within EMS systems is crucial to addressing these challenges. Secondary triage systems, in which clinical professionals reassess calls through phone interviews, have proven effective in reducing overtriage rates. Studies from other jurisdictions have shown that this approach can significantly reduce EMS workloads without compromising patient safety [25–27]. In Quebec, a nurse-led initiative focuses on P4 and P7 calls to redirect non-urgent cases to alternative care pathways, such as teleconsultation, alternative transportation, or clinic appointments. However, changes to priority definitions introduced in 2023 have reduced the number of calls eligible for secondary triage, which has diminished potential system benefits by approximately 622 cases (calls that have been upgraded from a non-urgent to an urgent priority). Re-evaluating these modifications could help restore the advantages of secondary triage while minimizing the risks of undertriage. A focus on specific chief complaints with high overtriage rates and the integration of clinical expertise into dispatch processes could better align resources with patient needs and improve overall EMS system performance. Additionally, implementing secondary triage systems to reassess urgent priorities assigned to specific chief complaints with known high overtriage rates could better align EMS resources with patient needs and improve overall system performance.

Artificial intelligence (AI)-powered tools represent a transformative opportunity to enhance EMS prioritization. Machine learning algorithms could analyze historical data to predict urgency levels more accurately, while predictive analytics could guide real-time resource deployment [28, 29]. When combined with secondary triage systems, these technologies could refine decision-making processes to reduce both overtriage and undertriage rates. For instance, AI could help identify patterns in high-frequency, non-urgent calls to help target interventions and reduce false positives. As the demand for EMS services continues to grow, and did so up to 8% between 2021 and 2023 largely due to demographic changes such as population aging, it is crucial for researchers and decision-makers to develop innovative solutions and alternative models of care.

This analysis underscores the importance of focusing on high-volume, high-overtriage call types to improve system performance. Evaluating the factors of overtriage in the emergency medical dispatch process offers a promising avenue for system enhancement. Additionally, leveraging interdisciplinary collaboration between EMS, clinical professionals, and AI researchers can lead to more effective and sustainable solutions. Future studies should prioritize the development of tailored interventions that align EMS responses with patient needs while mitigating the operational and safety risks associated with overtriage. Focusing on improving the prioritization performance relative to some high occurrence categories such as falls and sick persons could have a significant impact on the overall system efficiency.

### Limitations

This study has limitations that should be considered when interpreting the findings. First, the study is a retrospective observational one based on extracted data from three 5-month periods, which were selected due to data availability (convenience sampling). As a result, the findings might vary if data were collected during different times of the year due to the introduction of seasonal biases. Additionally, protocol changes in 2023 may have introduced bias in the evaluation of the overall performance of the system. However, these changes were also an opportunity to assess their impact on the prioritization system.

Second, overtriage and undertriage rates must be calculated using on-scene paramedic assessment and the need to transport patients using lights and sirens (immediate or urgent) or in a non-urgent manner. While the validity of this datapoint as a reference for assessing the urgency of the situation at dispatch may be arguable, we believe it is appropriate as it represents the first decision point after dispatch that can be reliably captured retrospectively for comparison. However, one limitation of this approach is the lack of clinical data to validate the true urgency of cases. Without such data, we cannot directly assess patient outcomes or compare paramedics’ transport decisions with emergency department final diagnosis and resource needs. Nonetheless, in our context, paramedics typically select urgent or immediate transport modes when they identify a potential risk of instability or time-sensitive conditions as per clinical protocols designed to ensure consistency across the province (Table 1). In some cases, prolonged delays before dispatch may contribute to an increase in condition urgency, even if the initial prioritization is appropriately lower. Accordingly, we argue that our overtriage rates are likely underestimated and our undertriage rates overestimated, which would make the solutions proposed in this study even safer.

Finally, it is important to acknowledge that patients cannot wait indefinitely for assistance even if they lack the criteria for time-sensitive conditions. For example, a patient who has fallen and is stable should still not be left waiting for hours. Thus, our study primarily advocates for improved prioritization to ensure that the right resource is dispatched to the right patient at the right time to enhance both system efficiency and patient care.

## Conclusions

This study reveals that the majority of urgent dispatches in our system were ultimately deemed non-urgent by paramedics, indicating a high overtriage rate. Modifications to priorities can significantly impact the balance between overtriage and undertriage, with important systemic consequences. While reducing undertriage may benefit specific patient groups with time-sensitive conditions, it is crucial to weigh these advantages against potential systemic effects, such as increased workload and resource strain. Future research should focus on establishing acceptable benchmarks of overtriage and undertriage for EMS systems. Additionally, further exploration is needed to assess the broader implications of high overtriage rates, particularly their impact on resource allocation, system efficiency, and patient outcomes in prehospital care.

## Electronic supplementary material

Below is the link to the electronic supplementary material.

Supplementary Material 1

Supplementary Material 2

Supplementary Material 3

## Data Availability

The data that support the findings of this study are not openly available due to reasons of sensitivity and are available from the corresponding author upon reasonable request.

## Abbreviations

AI: Artificial Intelligence
AUC: Area Under the Curve
BLS: Basic Life Support
CBD: Criteria Based Dispatch
CI: Confidence Interval
CTAS: Canadian Triage and Acuity Scale
EMS: Emergency Medical Services
FN: False Negative
FP: False Positive
MPDS: Medical Priority Dispatch System
NPV: Negative Predictive Value
PPV: Positive Predictive Value
ROC: Receiver Operating Characteristic
TN: True Negative
TP: True Positive
