# Supplementary material for: Performance measures of the medical priority dispatch system in an urban basic life support system
Source: Scand J Trauma Resusc Emerg Med. 2025 May 21;33:94. doi: 10.1186/s13049-025-01410-6 (PMC12096499; doi:10.1186/s13049-025-01410-6)
Supplement: Supplementary file 1 — Supplementary Material 1 [file 13049_2025_1410_MOESM1_ESM.docx]

**Supplementary material A: MPDS analysis**

Table A1 highlights the association between the MPDS code at call-taking and the paramedic assessment. The dataset shows a progression from priority Echo to Alpha regarding the proportion of calls considered non urgent/time sensitive by the paramedics. For example, it is possible to observe that over all calls identified as Echo at call-taking, 35.7% were deemed non urgent/time sensitive by the paramedics. This proportion reaches 62.7% and 77.1% for Delta and Charlie, respectively.

**Table A1 - Number of calls by MPDS code at call-taking and paramedic assessment**

| **MPDS code at call-taking** | **Paramedic assessment** | | **Total** |
| --- | --- | --- | --- |
|  | **Unstable or potentially unstable**  (Return transport mode: Immediate & Urgent) | **Stable**  (Return transport mode: Non-urgent) |  |
| **Echo** | 187 (64.3%) | 104 (35.7%) | 291 |
| **Delta** | 9,170 (37.3%) | 15,381 (62.7%) | 24,551 |
| **Charlie** | 6,341 (22.9%) | 21,398 (77.1%) | 27,739 |
| **Bravo** | 1,840 (11.1%) | 14,787 (88.9%) | 16,627 |
| **Alpha** | 1,949 (7.2%) | 24,942 (92.8%) | 26,891 |
| **Total** | 19,487 | 76,612 | 96,099 |

Percentages in parentheses are relative to the total for each MPDS letter code.

Table A2 presents the results for the MPDS system. Overall, the sensitivity is 90.0% (95% CI 89.6-90.4), along with an overtriage rate of 74.7% (95% CI 74.3-75) and a PPV of 25.3% (95% CI 25-25.7). Specificity is 32.6% (95% CI 32.2-32.9), while undertriage and NPV are 7.2% (95% CI 6.9-7.5) and 92.8% (95% CI 92.4-93.2), respectively. The accuracy and the AUC are 44.2% (95% CI 43.9-44.5) and 0.7028. However, it remains challenging to determine which system performs best overall, as critical thresholds for other performance metrics are not established and could be significant for EMS decision-making.

**Table A2 - Performance metrics for MPDS system**

| **Performance metric** | **MPDS** | | | **MPDS**  **(2021-2023)** |
| --- | --- | --- | --- | --- |
|  | **2021** | **2022** | **2023** |  |
| **Sensitivity** | 88.9% | 90.1% | 90.8% | **90%** |
| **Specificity** | 34.3% | 32.1% | 31.5% | **32.6%** |
| **Undertriage** | 7.7% | 7.5% | 6.7% | **7.2%** |
| **Overtriage** | 74.1% | 74.2% | 75.4% | **74.7%** |
| **NPV** | 92.3% | 92.5% | 93.3% | **92.8%** |
| **PPV** | 25.9% | 25.8% | 24.6% | **25.3%** |
| **Accuracy** | 45.5% | 44.1% | 43.2% | **44.2%** |

Results presented in Table A3 and Table A4 allow us to assess the consistency of performance across chief complaints and to identify specific codes with high rates of overtriage or undertriage. They indicate that abdominal pain (n=3,967), back pain (n=1,886), and psychiatric problems (n=3,441) were among the most frequent call types with high overtriage rates (greater than 90%), whereas allergic reactions (n=837), convulsions (n=1,857), diabetic problems (n=532), heart problems (n=1,876), overdose/poison (n=2,126), traffic incidents (n=3,644), and unconscious (n=7,750) showed the highest rates of undertriage (greater than 10%).

**Table A3 - Differences between dispatch priority and paramedic assessment across chief complaints**

| **Chief complaint (n)** | **Dispatch priority at call-taking % (n)** | | **Paramedic assessment % (n)  Urgent** | | **Paramedic assessment % (n)  Non Urgent** | |
| --- | --- | --- | --- | --- | --- | --- |
|  | **Urgent  (C, D, E)** | **Non Urgent**  **(A, B)** | **TP** | **FP** | **TN** | **FN** |
| Abdominal pain (3,967) | 52 (2,077) | 48 (1,890) | 10 (217) | 90 (1,860) | 93 (1,764) | 7 (126) |
| Allergic reaction (837) | 82 (689) | 18 (148) | 54 (374) | 46 (315) | 80 (119) | 20 (29) |
| Assault (489) | 94 (458) | 6 (31) | 14 (64) | 86 (394) | 100 (31) | 0 (0) |
| Back pain (1,886) | 39 (728) | 61 (1,158) | 5 (39) | 95 (689) | 99 (1,143) | 1 (15) |
| Breathing diff. (9,739) | 100 (9,739) | 0 (0) | 37 (3,568) | 63 (6,171) | NA (0) | NA (0) |
| Hazard exposure (114) | 100 (114) | 0 (0) | 39 (44) | 61 (70) | NA (0) | NA (0) |
| Cardiac arrest (103) | 87 (90) | 13 (13) | 73 (66) | 27 (24) | 100 (13) | 0 (0) |
| Chest pain (11,183) | 98 (10,984) | 2 (199) | 31 (3,373) | 69 (7,611) | 90 (179) | 10 (20) |
| Choking (371) | 67 (248) | 33 (123) | 39 (96) | 61 (152) | 92 (113) | 8 (10) |
| Convulsions (1,857) | 65 (1,208) | 35 (649) | 45 (547) | 55 (661) | 71 (459) | 29 (190) |
| Diabetic prob. (532) | 70 (370) | 30 (162) | 32 (119) | 68 (251) | 84 (136) | 16 (26) |
| Eye Problems (110) | 19 (21) | 81 (89) | 19 (4) | 81 (17) | 94 (84) | 6 (5) |
| Falls (14,380) | 65 (9,295) | 35 (5,085) | 11 (1,010) | 89 (8,285) | 93 (4,738) | 7 (347) |
| Headache (1,082) | 72 (776) | 28 (306) | 14 (109) | 86 (667) | 97 (296) | 3 (10) |
| Heart Problem (1,876) | 95 (1,789) | 5 (87) | 31 (556) | 69 (1,233) | 86 (75) | 14 (12) |
| Hemorrhage (3,758) | 87 (3,283) | 13 (475) | 14 (455) | 86 (2,828) | 95 (449) | 5 (26) |
| Overdose / Poison (2,126) | 98 (2,089) | 2 (37) | 31 (641) | 69 (1,448) | 84 (31) | 16 (6) |
| Pregnancy (409) | 97 (397) | 3 (12) | 47 (188) | 53 (209) | 100 (12) | 0 (0) |
| Psychiatric (3,441) | 48 (1,656) | 52 (1,785) | 9 (144) | 91 (1,512) | 96 (1,710) | 4 (75) |
| Sick Person (14,322) | 41 (5,855) | 59 (8,467) | 15 (895) | 85 (4,960) | 94 (7,987) | 6 (480) |
| Stroke (3,087) | 100 (3,084) | 0 (3) | 35 (1094) | 65 (1,990) | 100 (3) | 0 (0) |
| Traffic incidents (3,644) | 97 (3,538) | 3 (106) | 24 (833) | 76 (2,705) | 84 (89) | 16 (17) |
| Traumatic injuries (3,691) | 47 (1,721) | 53 (1,970) | 16 (279) | 84 (1,442) | 97 (1,906) | 3 (64) |
| Unconscious (7,750) | 69 (5,372) | 31 (2,378) | 32 (1,717) | 68 (3,655) | 85 (2,018) | 15 (360) |
| Unknown (966) | 100 (966) | 0 (0) | 23 (221) | 77 (745) | NA (0) | NA (0) |
| Palliative Care (3,868) | 57 (2,224) | 43 (1,644) | 32 (716) | 68 (1,508) | 93 (1,526) | 7 (118) |
| Pandemic (128) | 100 (128) | 0 (0) | 38 (49) | 62 (79) | NA (0) | NA (0) |

NA values not computed for lack of enough emergency calls of the specified category.

**Table A4 - Performance measures by MPDS category.**

| **Chief complaint (n)** | **Overtriage** | **Undertriage** | **Sensitivity** | **Specificity** | **PPV** | **NPV** | **Accuracy** |
| --- | --- | --- | --- | --- | --- | --- | --- |
| Abdominal pain (3,967) | 90% | 7% | 50% | 63% | 49% | 10% | 93% |
| Allergic reaction (837) | 46% | 20% | 59% | 93% | 27% | 54% | 80% |
| Assault (489) | 86% | 0% | 19% | 100% | 7% | 14% | 100% |
| Back pain (1,886) | 95% | 1% | 63% | 72% | 62% | 5% | 99% |
| Breathing diff. (9,739) | 63% | NA | 37% | 100% | 0% | 37% | NA |
| Hazard exposure (114) | 61% | NA | 39% | 100% | 0% | 39% | NA |
| Cardiac arrest (103) | 27% | 0% | 77% | 100% | 35% | 73% | 100% |
| Chest pain (11,183) | 69% | 10% | 32% | 99% | 2% | 31% | 90% |
| Choking (371) | 61% | 8% | 56% | 91% | 43% | 39% | 92% |
| Convulsions (1,857) | 55% | 29% | 54% | 74% | 41% | 45% | 71% |
| Diabetic prob. (532) | 68% | 16% | 48% | 82% | 35% | 32% | 84% |
| Eye Problems (110) | 81% | 6% | 80% | 44% | 83% | 19% | 94% |
| Falls (14,380) | 89% | 7% | 40% | 74% | 36% | 11% | 93% |
| Headache (1,082) | 86% | 3% | 37% | 92% | 31% | 14% | 97% |
| Heart Problem (1,876) | 69% | 14% | 34% | 98% | 6% | 31% | 86% |
| Hemorrhage (3,758) | 86% | 5% | 24% | 95% | 14% | 14% | 95% |
| Overdose / Poison (2,126) | 69% | 16% | 32% | 99% | 2% | 31% | 84% |
| Pregnancy (409) | 53% | 0% | 49% | 100% | 5% | 47% | 100% |
| Psychiatric (3,441) | 91% | 4% | 54% | 66% | 53% | 9% | 96% |
| Sick Person (14,322) | 85% | 6% | 62% | 65% | 62% | 15% | 94% |
| Stroke (3,087) | 65% | 0% | 36% | 100% | 0% | 35% | 100% |
| Traffic incidents (3,644) | 76% | 16% | 25% | 98% | 3% | 24% | 84% |
| Traumatic injuries (3,691) | 84% | 3% | 59% | 81% | 57% | 16% | 97% |
| Unconscious (7,750) | 68% | 15% | 48% | 83% | 36% | 32% | 85% |
| Unknown (966) | 77% | NA | 23% | 100% | 0% | 23% | NA |
| Palliative Care (3,868) | 68% | 7% | 58% | 86% | 50% | 32% | 93% |
| Pandemic (128) | 62% | NA | 38% | 100% | 0% | 38% | NA |

NA values not computed for lack of enough emergency calls of the specified category.
