# Supplementary material for: Performance measures of the medical priority dispatch system in an urban basic life support system
Source: Scand J Trauma Resusc Emerg Med. 2025 May 21;33:94. doi: 10.1186/s13049-025-01410-6 (PMC12096499; doi:10.1186/s13049-025-01410-6)
Supplement: Supplementary file 2 — Supplementary Material 2 [file 13049_2025_1410_MOESM2_ESM.docx]

**Supplementary material C: Changes in MPDS**

From June 2023, some changes were implemented with the MPDS code and priorities, based on the recommendations of the national committee of experts. The 2021 version of MPDS contained 1,526 codes while the 2023 version included 1,534. The priorities were modified for 126 different codes (8.3%). Of these 102 codes (8.3%) were upgraded (now more urgent) and 24 were downgraded (1.6%) (now less urgent). The upgraded priorities affected 6,263 calls which represent 16% of the total volume of calls in the 2023 period. The effect of these changes are visible in Table C1 with a decreased proportion of P7 and P3 moving towards a higher proportion of P4 and P1.

**Table C1 - Net changes in Priorities and MPDS codes between 2021 and 2023.**

| **MPDS\Priority** | **P0** | **P1** | **P3** | **P4** | **P7** |
| --- | --- | --- | --- | --- | --- |
| **Echo** | 3 | -3 | 0 | 0 | 0 |
| **Delta** | 12 | -5 | -7 | 0 | 0 |
| **Charlie** | 0 | 29 | -29 | 0 | 0 |
| **Bravo** | 7 | -2 | -5 | 0 | 0 |
| **Alpha** | 0 | 6 | -3 | 5 | -8 |
