# Supplementary material for: Performance measures of the medical priority dispatch system in an urban basic life support system
Source: Scand J Trauma Resusc Emerg Med. 2025 May 21;33:94. doi: 10.1186/s13049-025-01410-6 (PMC12096499; doi:10.1186/s13049-025-01410-6)
Supplement: Supplementary file 3 — Supplementary Material 3 [file 13049_2025_1410_MOESM3_ESM.docx]

# Supplementary material B: P-system excluding P3

Practitioners recognize that P3 dispatches, which account for over 40% of total call volume, introduce significant heterogeneity and are difficult to classify definitively as either urgent or non-urgent. To assess their impact on system performance, we conducted all analyses excluding P3 calls from the dataset. This approach allowed us to evaluate system performance considering only P0, P1, P4, and P7. Table B1 present the results for 2021, 2022, 2023, and overall. Since P3 dispatches are frequently overtriaged, their removal from the dataset increases specificity to 42.4% (95% CI: 41.9–42.9), PPV to 33.8% (95% CI: 33.3–34.2), and accuracy to 54.5% (95% CI: 54.1–54.9), while reducing the overtriage rate to 66.2% (95% CI: 65.8–66.7). Sensitivity slightly decreases to 92.5% (95% CI: 92–93), while other performance measures remain unchanged.

**Table B1 - Performance metrics for the P-system removing P3.**

| **Performance metric** | **P-system** | | | **P-system (2021-2023)** |
| --- | --- | --- | --- | --- |
|  | **2021** | **2022** | **2023** |  |
| **Sensitivity** | 90.5% | 91.9% | 94.3% | **92.5%** |
| **Specificity** | 48.1% | 44.8% | 36.7% | **42.4%** |
| **Undertriage** | 5.9% | 5.5% | 4.7% | **5.4%** |
| **Overtriage** | 64.3% | 65.3% | 68% | **66.2%** |
| **NPV** | 94.1% | 94.5% | 95.3% | **94.6%** |
| **PPV** | 35.7% | 34.7% | 32% | **33.8%** |
| **Accuracy** | 58.3% | 56.21% | 50.5% | **54.5%** |
